# Beyond the black box in healthcare operations: a structured literature review and governance framework for explainable AI in clinical process intelligence systems

**DOI:** 10.3389/frai.2026.1864565

**Published:** 2026-10-02

**Authors:** Albert Adusei Brobbey, Narayan Bhosale, James Ackom

**Affiliations:** California InterContinental University, Sioux Falls, SD, United States

**Keywords:** algorithmic transparency, clinical process intelligence, CPI-XAI Framework, Explainable AI, health informatics, healthcare AI governance, IS Success Model, IT Capability Theory

## Abstract

**Background:**

Healthcare artificial intelligence has produced substantial diagnostic capabilities, yet clinical operations remain underserved by AI transparency research. Existing reviews of Explainable AI (XAI) in healthcare concentrate on diagnostic imaging, oncology, radiology, and clinical decision support. The governance needs of clinical process intelligence (CPI) systems, which support patient flow, resource allocation, throughput management, and care coordination, remain comparatively underdeveloped.

**Objective:**

This original research article reports a structured literature review with systematic search and screening, followed by framework development. It characterizes healthcare XAI research across application domains and introduces the CPI-XAI Governance Framework, a theory-informed model for operationalizing algorithmic transparency as an organizational information-systems capability in clinical process intelligence environments.

**Methods:**

We searched PubMed/MEDLINE, Scopus, Web of Science, IEEE Xplore, ACM Digital Library, and CINAHL for peer-reviewed studies published from January 2015 to April 2026. Screening and extraction used transparent procedures adapted from PRISMA flow-reporting practices. Two authors independently screened records and extracted domain, XAI method, setting, evaluation, and governance variables; disagreements were resolved by author adjudication. Framework development used a documented hybrid deductive-inductive coding pipeline, source-contribution rules, negative-case review, and an auditable evidence-to-framework mapping anchored in IT Capability Theory, the DeLone–McLean IS Success Model, implementation science, sociotechnical systems theory, and health AI governance guidance.

**Results:**

Of 187 included studies, 175 (93.6%) addressed diagnostic, disease-specific, imaging, or general clinical decision-support contexts, while operational and workflow-embedded AI accounted for 9 studies (4.8%). The primary result is descriptive: XAI research in healthcare remains concentrated in diagnostic domains, while explicit explainability-governance work for clinical operations is sparse within the search boundary. Five evidence gaps informed the framework: limited operational XAI evaluation standards, role-blind explanation design, weak causal and counterfactual support, poor workflow embedding, and absence of longitudinal XAI governance. A preliminary 0–15 scoring rubric and two hypothetical organizational assessments demonstrate how the framework can be applied analytically; they do not constitute external validation.

**Conclusion:**

Healthcare XAI remains concentrated in diagnostic and clinical decision-support contexts, while explicit explainability-governance research for operational AI systems remains thinly represented. The CPI-XAI Governance Framework addresses this gap by treating explainability as an organizational capability rather than a model-level technical feature. The framework is preliminary and theory-informed; it should be empirically validated before use as a measurement instrument.

## 1 Introduction

Healthcare AI has matured considerably. Machine learning models now flag sepsis risk before clinicians can, identify malignancies in radiological images with expert-level accuracy, and predict readmission rates at discharge. What has not kept pace is the organizational infrastructure to govern how these systems explain themselves to the people who depend on them.

Explainable AI (XAI) describes the methods and design principles that allow AI systems to communicate the reasoning behind their outputs in terms that human users can understand, verify, and act upon. The healthcare XAI literature is substantial. A 2025 meta-analysis synthesized 62 peer-reviewed studies of XAI in clinical decision support systems across radiology, oncology, neurology, and critical care. A 2026 structured literature review in Frontiers in Artificial Intelligence covered 36 empirical studies across imaging, diagnosis, and rehabilitation. A comprehensive 2025 review following PRISMA guidelines included 170 core references across clinical XAI domains (Salimparsa et al., 2025; Aravindkumar et al., 2026; Ratz et al., 2025; Freyer et al., 2024).

Diagnostic AI refers here to systems that support patient-level detection, classification, diagnosis, prognosis, or treatment advice. Operational AI refers to systems that support organization-level decisions such as patient flow, bed allocation, staffing, scheduling, throughput, laboratory routing, discharge coordination, or capacity management. The pattern across the XAI literature is consistent: diagnostic tasks dominate. By contrast, the clinical operational domain, where AI may influence patient throughput, staff allocation, bed management, ED flow, lab turnaround, and supply chain coordination, remains weakly represented in the XAI conversation.

This imbalance creates a governance gap with practical consequences. When a clinical process AI system recommends diverting surgical capacity, adjusting nurse-to-patient ratios, or flagging a bottleneck in laboratory processing, users need to understand the basis for the recommendation. Without explanation infrastructure, AI-driven operational decisions may be accepted or rejected on intuition rather than informed judgment.

Rationale, scope, objectives, and expected contribution. The rationale for this review is that operational AI can alter throughput, capacity, staffing, care coordination, and resource allocation, but healthcare XAI research has focused mainly on diagnostic and disease-specific tasks. The scope is restricted to peer-reviewed XAI literature from January 2015 to April 2026 and its implications for operational and workflow-embedded AI in clinical process intelligence (CPI) systems.

Objective 1: Map the distribution of healthcare XAI studies across clinical application domains.

Objective 2: Characterize the operational and workflow-embedded AI subset and its reported explanation-governance features.

Objective 3: Identify evidence gaps that justify a CPI-specific explainability-governance framework.

Objective 4: Propose a validation roadmap for the CPI-XAI maturity model as a preliminary organizational capability framework.

Expected impact: Addressing this gap can help hospitals, vendors, and governance committees move from generic model-transparency claims toward role-aware, workflow-embedded, auditable explanation practices for operational AI.

## 2 Background and theoretical foundations

### 2.1 Explainable AI: scope and core methods

XAI encompasses strategies and methodologies for constructing AI systems that allow end users to understand and interpret model outputs. The field emerged in response to the deployment of high-performing but opaque models, particularly deep learning architectures, in domains where errors carry serious consequences (Arrieta et al., 2020; Markus et al., 2021; Adadi and Berrada, 2018; Minh et al., 2022).

The major XAI method families include: (1) *post-hoc* attribution methods such as SHAP (SHapley Additive exPlanations) and LIME (Local Interpretable Model-agnostic Explanations); (2) visualization-based methods such as Grad-CAM and saliency maps for image-based tasks; (3) counterfactual explanation methods describing what minimal changes to input would alter model output; and (4) concept-based explanations and causal inference approaches, which identify causal rather than correlational relationships. These methods are categorized along two axes: model-specific versus model-agnostic, and local versus global explanatory scope (Ribeiro et al., 2016; Lundberg and Lee, 2017; Selvaraju et al., 2017; Wachter et al., 2017; Sadeghi et al., 2024; Muhammad and Bendechache, 2024).

### 2.2 XAI in healthcare: current state and known gaps

The clinical case for XAI is well established. AI adoption in healthcare is constrained by clinician trust, regulatory accountability requirements, and the ethical imperative that decisions affecting patient safety be traceable. Research has confirmed that the challenge is not simply generating clinician trust in AI, but calibrating that trust, since excessive trust in incorrect AI advice is as dangerous as unwarranted skepticism toward correct advice (Rosenbacke et al., 2024; Holzinger et al., 2019; Tonekaboni et al., 2019; Ghassemi et al., 2021; Kelly et al., 2019; Panch et al., 2019; Topol, 2019; Obermeyer et al., 2019).

Existing structured literature reviews consistently report three unresolved problems. First, XAI evaluation remains inconsistent: most studies report model accuracy, F1-score, and AUC, while explanation quality, including faithfulness, stability, and clinical usability, receives minimal empirical attention (Noor et al., 2025). Second, workflow integration is rarely examined: XAI systems are typically evaluated in controlled conditions, not tested for fit with real-time clinical workflows under operational pressure (Lin et al., 2025). Third, organizational governance for XAI is absent: there are no validated frameworks for how healthcare institutions should evaluate, deploy, or audit XAI properties of operational AI systems (Zhang et al., 2022; van der Velden et al., 2022; Haupt et al., 2025).

This gap also connects to human-computer interaction (HCI) and user-centered explanation design. HCI research emphasizes that explanations should be shaped by user goals, task context, decision authority, and the questions users actually need answered, rather than by algorithmic availability alone (Abdul et al., 2018; Miller, 2019; Liao and Varshney, 2021). This literature strengthens the rationale for role-aware explanation design in CPI systems: a charge nurse, bed manager, quality-improvement lead, and hospital executive may need different explanations for the same operational recommendation.

### 2.3 Clinical process intelligence (CPI)

Clinical Process Intelligence refers to the organizational capability to collect, integrate, analyze, and act on data generated by clinical operational workflows. Where clinical decision support systems address individual patient encounters, CPI operates at the system level: understanding why an emergency department runs at capacity on Tuesday evenings, why lab turnaround degrades under specific staffing patterns, or where surgical scheduling creates downstream bottlenecks in ICU capacity.

CPI systems increasingly embed machine learning components for anomaly detection, predictive modeling of throughput constraints, and automated resource deployment recommendations. These differ from diagnostic AI in important ways. Their outputs target managers and administrators, not individual clinicians. Their errors aggregate into institutional consequences. And their explanations must satisfy operational stakeholders who lack clinical AI training, a user population entirely absent from existing XAI healthcare literature (Chen et al., 2020; Nasef et al., 2025; Bayor et al., 2025; Pahlevani et al., 2024).

The CPI maturity model establishes five organizational capability dimensions: Responsiveness (R): real-time operational signaling; Adaptability (A): workflow adjustment capability; Predictive Capability (P): forward-looking operational modeling; Integration (I): cross-system embedding; and Developmental Learning (D): longitudinal organizational learning. These dimensions provide the structural scaffolding for the CPI-XAI Framework.

### 2.4 Theoretical foundations

This study draws on two established IS frameworks. Bharadwaj’s (2000) IT Capability Theory conceptualizes IT capability as a firm-level resource integrating IT infrastructure, human IT skills, and IT-enabled intangibles. Applied here, XAI is not a technical feature but an organizational capability requiring infrastructure (explanation generation systems), human skill (interpretation literacy), and institutional integration (governance protocols for explanation auditing and accountability).

The DeLone and McLean (2003) IS Success Model provides the evaluative structure: information quality (Are explanations accurate and timely?), system quality (Are explanation interfaces usable and reliable?), and net benefit (Do explanations improve operational decisions?). This translates XAI evaluation from purely technical metrics into IS governance constructs accessible to healthcare administration.

### 2.5 Implementation science and health AI governance positioning

CPI-XAI is positioned in relation to implementation science and health AI governance. CFIR explains determinants that shape whether innovations are adopted in particular settings. NASSS explains why technologies become hard to scale, spread, or sustain in complex health systems. i-PARIHS emphasizes evidence, context, recipients, and facilitation. Sociotechnical systems theory foregrounds interactions among people, workflow, technology, organizational policy, and external regulation (Damschroder et al., 2009; Greenhalgh et al., 2017; Harvey and Kitson, 2016; Sittig and Singh, 2010).

These frameworks are necessary but not sufficient for the specific problem addressed here: how healthcare organizations should govern explanation quality for operational AI systems. CPI-XAI therefore occupies a narrower role as an explanation-governance framework for clinical process intelligence.

Major health AI governance guidance, including WHO guidance on AI for health, the NIST AI Risk Management Framework, and FDA materials on AI-enabled device software functions, emphasizes lifecycle management, transparency, risk management, and trustworthy deployment. CPI-XAI complements these sources by translating explainability governance into five clinical process intelligence capabilities: timely rationale, role-aware explanation, causal and counterfactual support, workflow-embedded access, and longitudinal explanation-fidelity monitoring. A comparison is provided in Table 1 (World Health Organization, 2021; National Institute of Standards and Technology, 2023; U.S. Food and Drug Administration, 2025b; U.S. Food and Drug Administration, 2025a).

**Table 1:** Complementarity of CPI-XAI with implementation science and health AI Governance Frameworks.

Framework/guidance	Primary focus	Value for this manuscript	Complementarity and boundary
CFIR	Implementation determinants	Explains context, barriers, facilitators, and adoption conditions.	Use CFIR to diagnose determinants; use CPI-XAI to specify explanation-governance capabilities within the implementation.
NASSS	Complexity and non-adoption	Explains why health technologies fail to scale, spread, or sustain.	Use NASSS to assess scale and complexity; use CPI-XAI to assess operational explanation governance.
i-PARIHS	Evidence, context, recipients, facilitation	Useful for planning facilitated implementation of AI governance practices.	Use i-PARIHS to plan facilitation; use CPI-XAI to define XAI-specific assessment content.
Sociotechnical systems theory	People-workflow-technology interaction	Shows why explanations must fit work systems and accountability structures.	Use sociotechnical analysis to understand the work system; use CPI-XAI to operationalize five explanation capabilities.
WHO, NIST, FDA AI guidance	Lifecycle risk, transparency, safety, trustworthy AI	Supplies policy and regulatory expectations for AI governance.	Use authoritative guidance for lifecycle obligations; use CPI-XAI as a CPI-specific assessment aid, subject to local legal and safety review.
CPI-XAI	Organisational explainability capability in CPI	Specifies timely, role-aware, causal, embedded, and longitudinal explanation governance.	Narrower operational-XAI specificity, but preliminary and not empirically superior to established frameworks or guidance.

## 3 Methods

### 3.1 Review design and reporting position

This original research article is a structured literature review and framework development study. It should not be read as a meta-analysis, registered scoping review, intervention-effectiveness review, risk-of-bias synthesis, or certainty-of-evidence assessment. The purpose was descriptive characterization of a defined XAI literature corpus and development of a governance framework for clinical process intelligence. The review was not prospectively registered in PROSPERO; therefore, no registration number is claimed. A protocol specifying search scope, eligibility criteria, screening process, coding variables, and planned descriptive synthesis was finalized before extraction and is available from the corresponding author on reasonable request. PRISMA 2020 was used selectively as a reporting scaffold for identification, deduplication, screening, eligibility decisions, exclusion counts, and the flow diagram. Because the study did not conduct intervention-effectiveness synthesis, formal risk-of-bias appraisal, or certainty-of-evidence grading, it does not claim full compliance as a conventional systematic review (Page et al., 2020; Tricco et al., 2018; Arksey and O'Malley, 2005; Levac et al., 2010).

### 3.2 Search strategy

We searched six databases: PubMed/MEDLINE, Scopus, Web of Science Core Collection, IEEE Xplore, ACM Digital Library, and CINAHL. Searches were executed between 12 March 2026 and 18 April 2026. Database-specific search strategies are reported in Supplementary Table S1. All searches used three conceptual blocks: explainability, healthcare setting, and operational or decision-support scope.

- Block 1 (Explainability): “Explainable AI” OR “XAI” OR “Algorithmic Transparency” OR “Interpretable Machine Learning” OR “Model Explainability” OR “Black-box AI”
- Block 2 (Healthcare): “Healthcare” OR “Clinical” OR “Hospital” OR “Health System” OR “Medical” OR “Patient Care”
- Block 3 (Scope): “Process” OR “Operations” OR “Workflow” OR “Throughput” OR “Resource Allocation” OR “Care Coordination” OR “Decision Support” OR “Clinical Informatics” OR “Health IT”

Date limits were January 2015 to April 2026. Restrictions were English language and peer-reviewed journal articles or full conference proceedings. Grey literature, editorials, opinion pieces, preprints, and conference abstracts without full text were excluded. Deduplication was performed in Zotero and manually verified in Microsoft Excel using title, author, year, DOI, and venue fields.

The search strategy intentionally required an explainability-related term. This boundary improves alignment with the paper’s XAI-governance question but may miss operational AI studies in health services management, operations research, industrial engineering, scheduling, bed management, and patient-flow modelling that use predictive modelling or optimization language without XAI terminology. A *post hoc* PubMed/MEDLINE boundary check on 10 June 2026 compared this search boundary: a broad operational-healthcare AI query without the XAI block returned 5,913 records, while the comparable query retaining XAI/explainability terms returned 135 records. These counts were not screened, deduplicated, or merged into the primary corpus and should not be interpreted as an estimate of 5,778 excluded relevant studies. They instead illustrate the likely direction and scale of vocabulary sensitivity: the wider operational AI field is much larger than the literature that explicitly labels explainability as a study focus.

A future search extension should use a two-stage strategy: first, broad operational AI terms without an XAI filter across health, engineering, operations-research, and management databases; second, targeted coding for explanation, transparency, accountability, contestability, and governance features whether or not authors use the label XAI. Citation chaining, expert nominations, multilingual searches, and structured searches of hospital, regulator, standards-body, procurement, and vendor materials could then test how grey and local evidence changes the framework.

This boundary is methodologically important. The review should not be read as a census of all operational AI in healthcare. Non-XAI operational AI studies in operations research, health systems engineering, scheduling, bed management, and patient-flow modelling are treated as adjacent contextual literature rather than as evidence in the primary corpus. This avoids conflating the existence of operational AI with the narrower question addressed here: whether operational AI systems are accompanied by explicit explainability and governance evidence.

### 3.3 Study selection and inter-rater reliability

Two authors independently screened all records at title/abstract stage, followed by full-text review for all potentially eligible studies. Disagreements were resolved through discussion; where consensus was not reached, the remaining author adjudicated. Screening decisions, exclusion reasons, and extracted variables were recorded in a structured spreadsheet.

Inter-rater reliability was assessed using Cohen’s kappa (*κ*) at both screening stages. At title/abstract screening, *κ* = 0.84, indicating strong agreement. At full-text review, *κ* = 0.88, indicating near-perfect agreement. These values exceed the *κ* ≥ 0.80 threshold recommended for structured literature review methodology (Landis and Koch, 1977).

### 3.4 Inclusion and exclusion criteria

See Table 2.

**Table 2:** Inclusion and exclusion criteria for study selection.

Dimension	Inclusion criteria	Exclusion criteria
Study design	Empirical, SLR, or framework studies with defined methodology	Editorials, opinion pieces, commentaries without empirical grounding
Context	Healthcare, clinical, or health system AI deployment settings	Laboratory-only, simulation environments, or non-clinical domains
XAI component	Explainability is a defined research variable or primary outcome	Pure algorithm papers with no clinical deployment or governance context
Outcomes reported	Clinical, organizational, workflow, or IS-level outcomes documented	Studies reporting only model accuracy metrics (AUC, F1) without governance context
Language	English	All other languages
Publication period	January 2015 to April 2026	Pre-2015

### 3.5 Data extraction

A standardized data extraction form captured study citation, year, country or region, clinical domain, AI model type, XAI method(s), user role, setting, study design, evaluation approach, governance feature, and whether the AI application was diagnostic, therapeutic, operational, workflow-embedded, or multidomain. A coding manual with operational definitions is provided in Supplementary Table S2. The nine operational/workflow studies are summarized in Table 3.

**Table 3:** Expanded characteristics of the operational/workflow AI subset (*n* = 9).

ID	Problem/setting	AI method	XAI type	Evaluation	Recipients	Role-specific?	Governance feature	Evidence context
OS1	ED patient-flow prediction	Supervised ML	SHAP/feature importance; *post-hoc*	Retrospective case study	Charge nurse; operations lead	Partial	Limited role-specific rationale	Retrospective
OS2	ED queue/crowding support	Predictive model	Feature attribution; *post-hoc*	Prototype evaluation	Operations manager	No	Workflow rationale partially reported	Prototype
OS3	Inpatient bed management	Prediction/optimization	Custom explanation layer; mixed	Single-institution implementation	Bed manager	Partial	Operational variables exposed	Implementation
OS4	Surgical scheduling/resources	Regression/ML	Coefficients or SHAP; *post-hoc*	Retrospective model evaluation	Administrator	No	Resource rationale available	Retrospective
OS5	Lab turnaround prediction	Supervised ML	Feature importance; *post-hoc*	Observational study	QI lead	No	Bottleneck drivers displayed	Observational
OS6	Discharge/transfer support	Rule-based/explainable model	Built-in rule rationale	Pilot deployment	Care coordinator	Partial	Care-team rationale included	Pilot
OS7	Staffing demand forecast	Forecasting model	Feature attribution; *post-hoc*	Simulation/retrospective validation	Nurse manager	No	Staffing drivers exposed	Simulation/retrospective
OS8	Operational risk stratification	Risk model	SHAP/partial dependence; *post-hoc*	Architecture/review study	Operations executive	No	XAI secondary to architecture	Framework/review
OS9	Workflow prediction/recommendation	Mixed AI	Mixed XAI	Framework/review study	Mixed operational users	Not reported	Limited governance discussion	Framework/review

A fuller extraction table is provided in the Supplementary workbook.

Because the review question was a domain-mapping and governance-gap question rather than an intervention-effectiveness question, study quality was not used to weight the domain counts. However, reviewers recorded whether each included study had a defined methodology, deployment or evaluation context, XAI component, and reported outcome. The absence of a full risk-of-bias synthesis is retained as a limitation. For future updates, the Mixed Methods Appraisal Tool (MMAT) would be appropriate because the corpus includes quantitative empirical studies, qualitative/user studies, mixed-methods evaluations, reviews, and framework papers (Hong et al., 2018).

#### 3.5.1 Source-contribution rules

Included publications were not treated as equally probative for framework construction. Empirical operational or workflow studies supplied direct evidence about users, interfaces, evaluation practices, and governance omissions in CPI settings. Empirical diagnostic or clinical decision-support studies supplied transferable XAI constructs but were not treated as evidence that those constructs are feasible in operational workflows. Review and framework papers were used to identify candidate concepts and compare conceptual coverage, while theory and governance guidance supplied deductive anchors and normative criteria rather than empirical validation. Study type therefore determined the role a source could play in the evidence chain; no numerical quality weight or vote count was used to imply that heterogeneous designs were commensurable.

For each proposed CPI-XAI dimension, retention required triangulation across at least two of three inputs: a recurrent observation in the operational subset, a transferable XAI requirement in the broader corpus, and an established IS, implementation, sociotechnical, or governance construct. A dimension was not retained solely because it appeared in a review or conceptual paper. This contribution rule improves methodological rigor while preserving the descriptive purpose of the review; it is not a substitute for formal risk-of-bias appraisal or external framework validation.

### 3.6 Descriptive analysis of domain distribution

The primary synthesis is descriptive. Domain counts and Wilson 95% confidence intervals were calculated for each mapped application domain. The main text does not rely on an inferential goodness-of-fit test because there is no theoretical reason to expect a uniform distribution of XAI research across clinical domains. An exploratory chi-square calculation is retained only in the Supplementary workbook as a numerical sensitivity descriptor. The substantive interpretation rests on observed proportions, especially the finding that operational/workflow AI accounted for 9 of 187 included studies (4.8%).

### 3.7 Framework development

The CPI-XAI Governance Framework was developed using hybrid deductive-inductive evidence-to-framework mapping. Deductive parent codes were specified from CPI capability domains, IT Capability Theory, the DeLone–McLean IS Success Model, implementation science, and sociotechnical systems theory. Inductive subcodes were added when repeated observations in the extracted studies were not adequately represented by those parent codes. For example, absent or undifferentiated recipient fields informed the role-blind explanation code; explanations reported outside the operational interface informed poor workflow embedding; and absence of post-update explanation monitoring informed the longitudinal-governance gap. Table 4 presents the derivation matrix linking evidence gaps to framework dimensions.

**Table 4:** Evidence-to-framework derivation matrix linking review findings to CPI-XAI dimensions.

Evidence gap	CPI-XAI dimension	IT capability construct	D&M construct	Framework requirement
Limited operational XAI standards	Responsiveness	Infrastructure capability	Information quality	Timely traceable rationale at point of operational decision
Role-blind explanation design	Adaptability	Human capability	System quality	Role-aware explanation depth and format
Weak causal and counterfactual support	Predictive capability	Infrastructure capability	Information quality	Counterfactual and causal explanation capability
Poor workflow embedding	Integration	Institutional integration capability	System quality	Explanation access inside operational workflow
No longitudinal XAI governance	Developmental learning	Institutional integration capability	Net benefit	Revalidation and learning loop for explanation quality

Framework development in this manuscript did not include an external Delphi panel, field validation, or stakeholder consensus process. The author team reviewed the coding logic and evidence-to-framework mapping internally, and the proposed empirical validation roadmap is intended to test, refine, and externally validate the framework before it is used as a formal assessment instrument.

Framework generation proceeded through four steps. First, the author team coded each included study for application domain, XAI method, setting, user role, evaluation approach, workflow embedding, and governance feature. Second, extracted governance features and absences were clustered into candidate evidence gaps using constant comparison across studies and the operational subset. Third, candidate gaps were mapped to CPI domains and IS constructs that could support organizational capability development. Fourth, the resulting dimensions were reviewed by the authors for conceptual non-overlap, operational relevance, negative or contradictory examples, and practical assessability. Disagreements were resolved through discussion and revision of code definitions. No separate reliability coefficient was calculated for this interpretive framework-coding stage; the reported kappa values apply to study selection. No external Delphi panel, stakeholder workshop, or psychometric validation was conducted in this version, so the framework remains preliminary and theory-informed.

#### 3.7.1 Framework coding and evidence-to-framework audit trail

1. Unit of coding: one included study was the primary unit; multiple reported systems or user groups were recorded in linked rows rather than collapsed.
2. Codebook control: deductive parent codes, inductive subcodes, inclusion and exclusion rules, and positive, negative, and borderline examples were versioned in the coding manual (Supplementary Table S2).
3. First-cycle coding: reviewers recorded setting, user role, workflow location, explanation type, timing, evaluation approach, governance feature, and explicit absence or non-reporting. Absence was coded only when the relevant feature was within the study’s stated scope; silence outside scope was coded as not reported rather than as evidence of non-existence.
4. Second-cycle synthesis: similar codes were clustered into candidate governance gaps using constant comparison. Each cluster retained links to study identifiers and verbatim or closely paraphrased evidence notes in the extraction workbook.
5. Framework mapping: candidate gaps were mapped to CPI capability domains and IS constructs only when the conceptual fit, operational consequence, and observable organizational evidence could be stated. Table 4 reports the condensed mapping chain.
6. Challenge review: the authors examined contradictory cases, overlapping dimensions, and alternative placements. Decisions to merge, retain, rename, or reject a candidate dimension were recorded in a decision log; disagreements were resolved by consensus and codebook revision.

##### 3.7.1.1 Reproducibility boundary

The screening kappa values do not apply to this interpretive framework-coding stage. The procedure and mapping are reproducible in the sense that the coding unit, rules, decision sequence, and audit trail are specified; independent recoding, intercoder agreement for the framework codes, stakeholder validation, and field testing remain outstanding.

## 4 Results

### 4.1 Search results and screening flow

Figure 1 presents the search and screening flow diagram. The initial database search returned 2,847 records across six databases. After duplicate removal (*n* = 634), 2,213 records underwent title and abstract screening. Title/abstract screening excluded 1,891 records (Cohen’s kappa = 0.84). Full-text review was conducted on 322 studies, yielding 187 studies in the final analytical corpus (Cohen’s kappa = 0.88 at full-text stage) (Haddaway et al., 2022).

**Figure 1:**
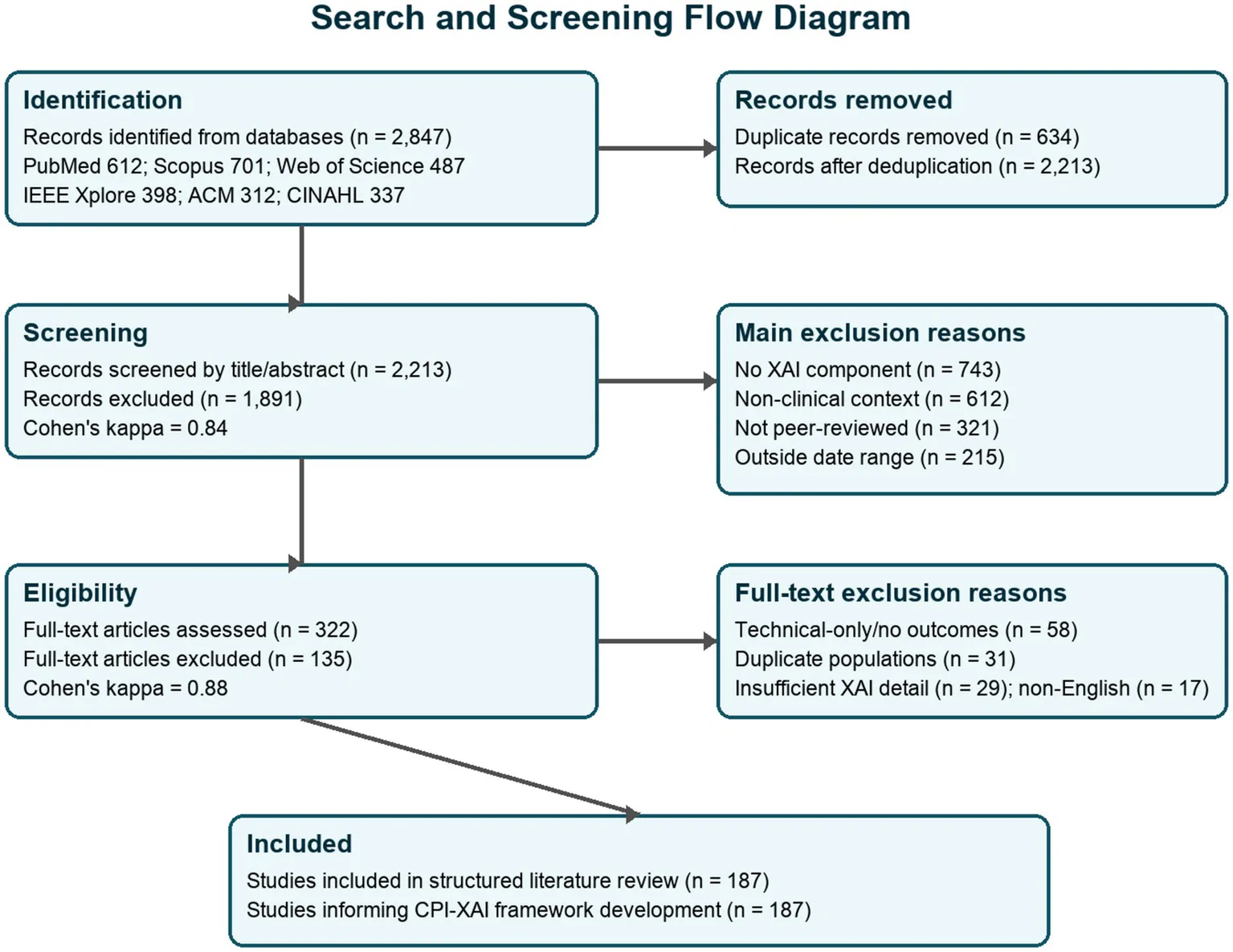
Search and screening flow diagram for study identification, screening, eligibility assessment, and inclusion.

### 4.2 Domain distribution: the diagnostic concentration

Table 5 presents the distribution of 187 included studies across clinical application domains. The concentration in diagnostic, disease-specific, imaging, and general clinical decision-support contexts is substantial: 175 studies (93.6%) fall into these categories. Only 9 studies (4.8%) examine operational or workflow-embedded AI systems.

**Table 5:** Distribution of XAI healthcare studies across clinical application domains (*n* = 187).

Clinical application domain	*n*	%	95% CI	Primary XAI methods
Diagnostic imaging (radiology, pathology)	74	39.6%	[32.7, 46.8%]	Grad-CAM, SHAP, attention mechanisms
Oncology/cancer detection	38	20.3%	[14.9, 26.9%]	SHAP, LIME, Grad-CAM
Neurology/brain imaging	24	12.8%	[8.5, 18.5%]	Grad-CAM, saliency maps
Cardiology/ECG analysis	18	9.6%	[5.9, 14.9%]	SHAP, integrated gradients
General clinical decision support	21	11.2%	[7.2, 16.8%]	LIME, SHAP, rule-based
Clinical operations/workflow AI	9	4.8%	[2.3, 9.0%]	SHAP (partial), custom
Other/multidomain	3	1.6%	[0.4, 4.6%]	Mixed
Total	187	100%		

95% CIs calculated using Wilson score method.

The Other/Multidomain category contained three studies (1.6%). These studies crossed more than one clinical area or discussed XAI at a general healthcare level without a dominant operational workflow focus. They were retained as a separate category to avoid artificially inflating either the diagnostic or operational counts.

### 4.3 Domain concentration and operational underrepresentation

The important result is descriptive. Operational and workflow-embedded AI accounted for 9 of 187 included studies (4.8%), while diagnostic, disease-specific, imaging, and general clinical decision-support contexts accounted for 175 studies (93.6%). This pattern supports a bounded claim: operational AI exists in healthcare, but explicit explainability-governance research for operational AI remains weakly represented within an XAI-filtered corpus.

Figure 2 visualizes the diagnostic concentration and operational underrepresentation that motivate the CPI-XAI framework.

**Figure 2:**
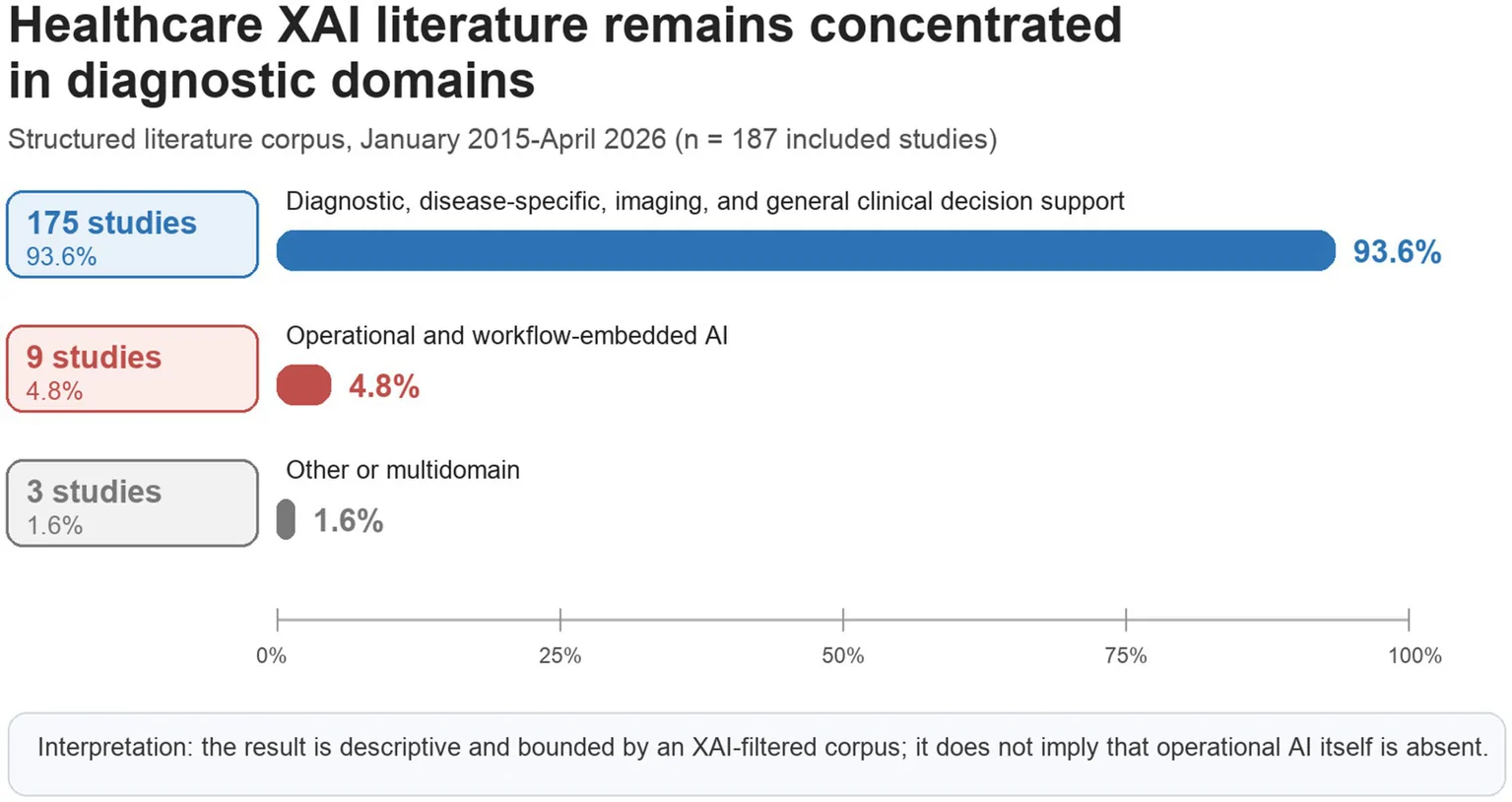
Visual contrast between diagnostic and operational XAI coverage in the included healthcare XAI corpus (*n* = 187).

A *post hoc* PubMed/MEDLINE boundary check conducted on 10 June 2026 illustrates this search-boundary issue. A broad operational-healthcare AI query without the XAI filter returned 5,913 records, whereas a comparable query retaining XAI and explainability terms returned 135 records. These counts were not screened or merged into the primary corpus, but they reinforce the interpretation that operational AI is a broader field than operational XAI governance. The framework therefore addresses the latter problem: the governance of explainability for operational AI systems.

### 4.4 Characterization of operational AI studies

The nine studies addressing clinical operations or workflow AI were examined in detail and are summarized in Table 3, with fuller extraction fields in the Supplementary workbook. Six of nine primarily used *post-hoc* feature-attribution or model-importance approaches; only three provided partial role-specific explanation design, five provided none, and one did not report it. Two studies described pilot or implementation contexts, while the remainder were retrospective, prototype, simulation, observational, architecture, or review-based. No study proposed a CPI-specific governance maturity framework or validated an operational explanation-quality instrument. Across settings, the recurring limitations were weak recipient differentiation, limited workflow-native explanation access, sparse accountability mechanisms, and little longitudinal testing after model or workflow change. These frequencies are descriptive of a small, heterogeneous subset and are not treated as population estimates.

Because the operational/workflow subset contains only nine studies, conclusions about operational XAI governance should be interpreted as gap-identification evidence rather than as a mature evidence base. The subset is sufficient to show limited coverage, heterogeneity of methods, and weak governance reporting, but not sufficient to estimate prevalence, effectiveness, or implementation success across healthcare operations.

### 4.5 Five evidence gaps informing framework development

#### 4.5.1 Gap 1: absence of operational XAI evaluation standards

Diagnostic XAI has developed reasonably consistent evaluation approaches, including fidelity measures, stability measures, and clinician usability assessments. Operational AI has none. There is no validated instrument for assessing whether an operational AI explanation is adequate for the organizational decision it supports. Existing XAI evaluation metrics from diagnostic contexts do not map cleanly to the needs of administrators, charge nurses, or quality improvement officers making system-level decisions (Agrawal et al., 2025).

#### 4.5.2 Gap 2: role-blind explanation design

Clinical operations involves multiple distinct user roles with different decision authority, accountability structures, and information needs. Published literature has acknowledged this user heterogeneity problem in diagnostic AI contexts but has not addressed it for operational AI users. An emergency department charge nurse and a hospital CFO evaluating the same AI-generated operational recommendation require fundamentally different explanation formats, granularity levels, and temporal framing, a design distinction absent from current XAI healthcare literature.

### 4.6 Role-specific operational requirements

Role-aware explanation is not simply a change in display length. A charge nurse may need current queue drivers, patient-flow bottlenecks, uncertainty, immediate actions, and escalation thresholds. A nurse manager may need shift-level demand, skill-mix implications, workload distribution, fairness across teams, and counterfactual staffing options. A quality-improvement officer may need aggregate trends, subgroup performance, audit trails, override patterns, and links to process outcomes. A hospital executive may need strategic capacity, financial, compliance, equity, and reputational implications rather than model-level feature weights. CMIOs, CIOs, and AI governance committees additionally require model version, data lineage, explanation fidelity, drift, access logs, and evidence that overrides and incidents are reviewed.

Operational XAI evaluation should therefore extend beyond diagnostic fidelity metrics. Candidate criteria include explanation timeliness, actionability, role fit, counterfactual usefulness, workflow accessibility, traceability to model and data versions, stability after updates, subgroup fairness, escalation support, and whether explanations improve calibrated reliance without increasing delay or cognitive burden (Singhal et al., 2024).

#### 4.6.1 Gap 3: no organizational maturity model for XAI governance

IS research has produced robust maturity models for IT governance, EHR adoption, and clinical informatics capability. The reviewed literature did not identify a CPI-specific maturity model for XAI governance in operational healthcare AI. Healthcare organizations therefore lack a validated instrument to assess their XAI readiness, identify governance deficiencies, or sequence capability-development investments. This gap motivates the CPI-XAI Framework.

#### 4.6.2 Gap 4: poor workflow embedding

Operational explainability is valuable only when it is available at the point of operational decision-making. The operational/workflow subset showed limited evidence that explanations were embedded inside EHRs, bed-management systems, staffing dashboards, scheduling platforms, or operational command-centre interfaces. Explanations were often reported as model outputs, research artefacts, or retrospective analytic descriptions rather than workflow-native governance resources.

#### 4.6.3 Gap 5: no longitudinal explanation governance

The reviewed operational studies rarely addressed whether explanation quality remains stable after model retraining, workflow redesign, seasonality shifts, data-source changes, or changes in hospital demand. This is important because operational AI systems may remain apparently functional while the meaning, fidelity, or actionability of their explanations drifts over time. CPI-XAI therefore includes developmental learning as a governance dimension.

## 5 The CPI-XAI Governance Framework

### 5.1 Framework overview

The CPI-XAI Framework is a five-dimension governance model for XAI capability in clinical process intelligence environments. Each dimension corresponds to one CPI capability domain and specifies the XAI governance requirements, evaluation constructs, and organizational capabilities necessary for responsible AI deployment in that domain. The framework retains the CPI model’s RAPID capability dimensions: Responsiveness, Adaptability, Predictive Capability, Integration, and Developmental Learning.

The framework is grounded in Bharadwaj’s IT Capability Theory, treating each CPI-XAI dimension as an organizational IS capability rather than a technical feature of individual AI models, and structured using DeLone–McLean IS Success constructs, making it evaluable using established IS metrics accessible to healthcare governance practitioners.

Figure 3 summarizes how the five CPI-XAI dimensions convert the evidence gaps identified in the review into organizational governance capabilities.

**Figure 3:**
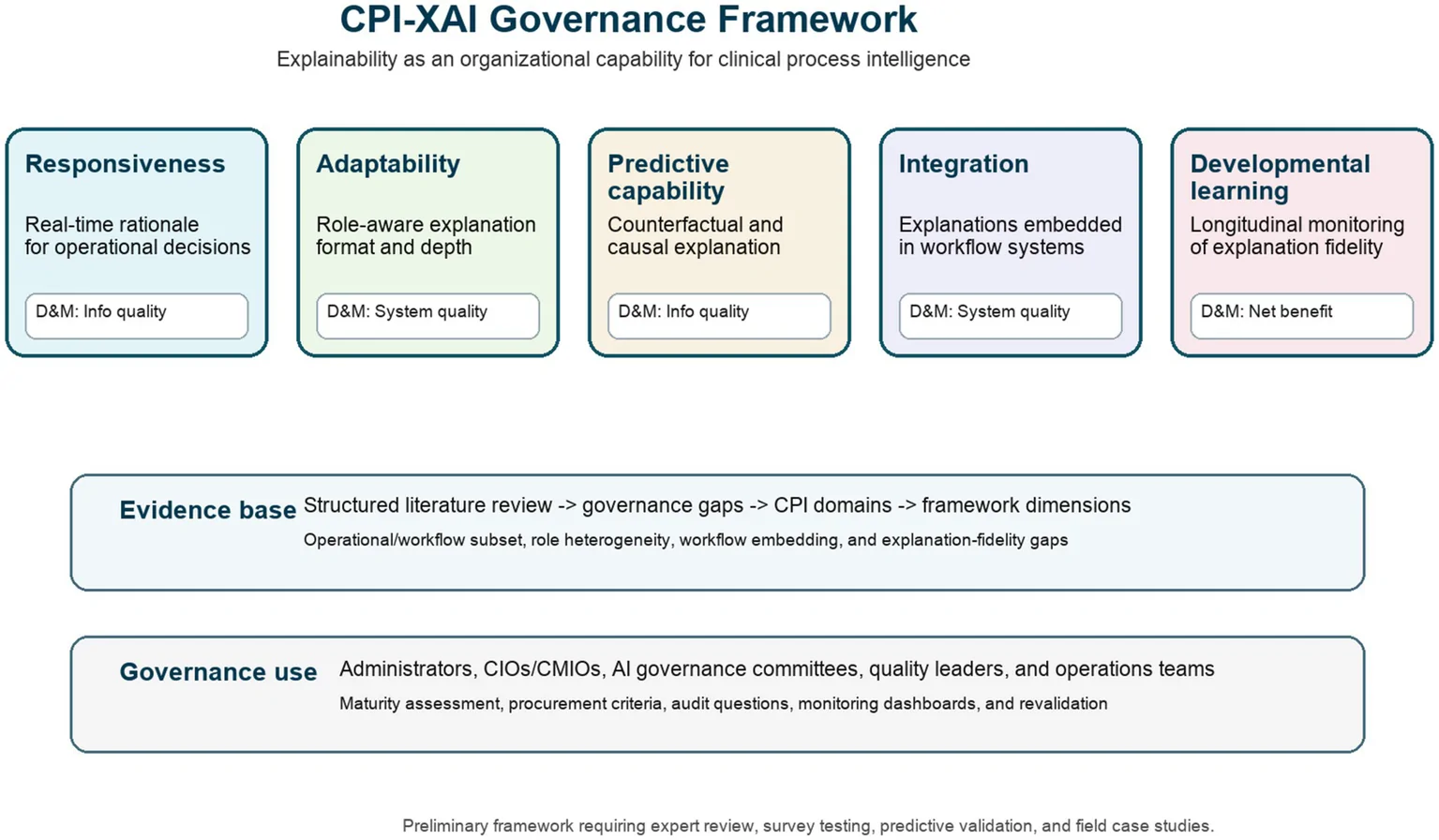
Visual summary of the CPI-XAI Governance Framework and its evidence-to-capability logic.

### 5.2 Framework dimensions

Table 6 shows CPI-XAI Framework dimensions mapped to CPI domains, DeLone–McLean IS success constructs, and Bharadwaj IT capability categories.

**Table 6:** CPI-XAI Framework dimensions mapped to CPI domains, DeLone–McLean IS success constructs, and Bharadwaj IT capability categories.

Dimension	CPI domain	XAI layer	Key construct	D & M axis	IT capability
R—Responsiveness	Real-time operational signaling	Alert transparency	Explanation timeliness: AI alerts include traceable rationale at point of delivery	Info quality	Infrastructure
A—Adaptability	Contextual workflow adjustment	Role-aware explanation delivery	User-role calibration: explanation depth and format match recipient decision authority	System quality	Human skill
P—Predictive	Forward-looking operational modeling	Causal vs. correlational clarity	Counterfactual availability: system can answer ‘what if X changes’ questions	Info quality	Infrastructure
I—Integration	Cross-system embedding	Workflow-embedded explainability	EHR and operational dashboard compatibility: explanations accessible within existing workflows	System quality	Inst. integration
D—Developmental	Longitudinal organizational learning	Explanation fidelity over time	Model drift accountability: explanation accuracy re-validated after model retraining	Net benefit	Inst. integration

### 5.3 The CPI-XAI Maturity Scale

Each CPI-XAI dimension is assessed across four preliminary maturity levels, constituting a proposed CPI-XAI Maturity Scale. The scale is intended as a conceptual diagnostic tool for future validation, not as a validated psychometric instrument in its present form:

An organization at Level 4 across all five CPI-XAI dimensions would approach what this framework terms XAI Operational Sovereignty. This term is used as an aspirational design goal: the capacity to deploy, monitor, and continuously improve the explainability of clinical process AI systems in ways that are role-appropriate, auditable, and integrated into operational workflows. The manuscript does not claim that this capability has been empirically validated or that Level 4 maturity produces superior organizational outcomes without further testing (see Table 7).

**Table 7:** Preliminary CPI-XAI Maturity Scale.

Maturity level	Label	Description	Governance implication
Level 1	Absent	No XAI capability in this dimension	Critical governance gap; AI outputs cannot be traced or audited
Level 2	Reactive	XAI exists but is generated *post-hoc* without organizational integration	Explanations available on request but not embedded in decision workflows
Level 3	Governed	XAI is embedded in workflows with defined accountability structures	Explanations are systematically generated, role-appropriate, and auditable
Level 4	Optimizing	Continuous evaluation and improvement of explanation quality with user feedback loops	XAI governance is a measured organizational capability with improvement targets

Level 4 represents an aspirational explainability-governance target rather than a validated maturity endpoint.

### 5.4 Preliminary operationalization indicators

To demonstrate how the conceptual maturity scale could become an assessment instrument, Table 8 provides preliminary candidate items and observable evidence. These items are illustrative, not validated. Future Delphi review should test wording, relevance, feasibility, and whether documentary evidence should be required in addition to self-report ratings.

**Table 8:** Preliminary CPI-XAI assessment items and evidence indicators.

Dimension	Illustrative assessment item	Observable evidence	Maturity progression
Responsiveness	Operational AI alerts provide a timely rationale linked to the current decision.	Timestamped explanation logs; alert rationale; escalation records	Absent → available on request → standardized at decision point → timeliness monitored and improved
Adaptability	Explanation depth and format are configured for each user’s decision authority and workflow.	Role profiles; interface variants; role-based user testing	One format → *ad hoc* tailoring → governed role profiles → continuously evaluated role fit
Predictive capability	Users can inspect uncertainty, controllable drivers, and relevant counterfactual scenarios.	Uncertainty displays; scenario panels; documented action thresholds	No causal support → occasional scenarios → standardized counterfactuals → outcome-linked validation
Integration	Explanations are accessible within the operational system where the decision is made.	EHR/dashboard integration; access logs; workflow usability tests	External report → linked tool → workflow-native access → monitored use and redesign
Developmental learning	Explanation fidelity is re-evaluated after model, data, or workflow change.	Versioned validation; drift alerts; reapproval and incident-review records	No review → incident-driven review → scheduled revalidation → continuous monitoring and learning

### 5.5 Scoring rubric and decision rules

For pilot use, each dimension is rated from 0 to 3, corresponding to the four maturity levels: 0 = absent, 1 = reactive, 2 = governed, and 3 = optimizing. Assessors should score documentary or system evidence rather than aspiration alone. The five ratings produce a provisional total from 0 to 15, but the dimension profile and lowest score must always accompany the total because compensation across dimensions can conceal a critical weakness (see Table 9).

**Table 9:** Provisional CPI-XAI scoring rubric. Scores are decision aids for pilot assessment and have not been psychometrically validated.

Score	Evidence threshold	Decision rule
0—Absent	No documented capability or observable evidence for the dimension.	Record as a critical governance gap.
1—Reactive	Ad hoc or *post-hoc* practice; evidence exists for isolated cases but no defined owner or standard workflow.	Prioritize ownership, minimum evidence, and repeatable procedure.
2—Governed	Documented process, accountable owner, workflow integration, and auditable evidence across routine use.	Monitor adherence and variation across roles or sites.
3—Optimizing	Governed capability plus measured performance, feedback, and improvement demonstrated across at least two review cycles.	Maintain continuous monitoring and outcome-linked improvement.

Provisional interpretation bands are 0–3 (nascent), 4–7 (reactive), 8–11 (governed-in-development), and 12–15 (advanced). Two safeguards limit overinterpretation: an organization with any dimension scored 0 cannot be classified above reactive, and no organization should claim an advanced profile unless all five dimensions score at least 2. These bands are heuristic and must be tested for reliability, construct validity, and predictive validity before benchmarking organizations.

### 5.6 Worked organizational assessments

Table 10 applies the rubric to two hypothetical organizational archetypes. The examples are analytic demonstrations based on the stated evidence rules, not observations of named hospitals, case-study findings, or evidence of clinical effectiveness.

**Table 10:** Worked hypothetical CPI-XAI assessments illustrating profile scoring, gating rules, and improvement priorities.

Hypothetical organization	R/A/P/I/D profile	Total and classification	Interpretation and priority
Community hospital piloting vendor bed-management AI	1/0/1/1/0	3/15; nascent (two zero-score gates)	Rationales are available only on request; there are no role profiles or post-update revalidation. Establish accountable owners, role-aware views, and change-triggered review before scale-up.
Integrated delivery system with workflow-native patient-flow AI	2/2/2/3/2	11/15; governed-in-development	Capabilities are documented and embedded, but most are not yet linked to repeated performance improvement. Test explanation outcomes and complete two review cycles before any optimizing score.

### 5.7 Applied example: ED patient flow AI

Consider a hospital using a CPI system to manage emergency department patient flow. The AI recommends diverting ambulances to a secondary facility based on current ED census, triage acuity distribution, and predicted discharge rates. The CPI-XAI Framework specifies the following governance requirements for responsible deployment of this recommendation:

- R (Responsiveness): the recommendation includes a real-time explanation of which operational variables drove it, presented at the moment the recommendation is generated, not available only upon retrospective query.
- A (Adaptability): the charge nurse receives operational specifics (current census breakdown, predicted discharge timing, triage severity distribution). The hospital administrator receives system-level trend data (weekly throughput patterns, staffing correlation with diversion events).
- P (Predictive): the system answers counterfactual questions: ‘What would the recommendation be if we reassigned two nurses from triage to the fast-track area?’ This is causal explainability, not merely feature attribution.
- I (Integration): explanations are accessible within the existing EHR and ED management dashboard, not in a separate analytics portal requiring additional navigation steps under time pressure.
- D (Developmental): when the AI model retrains on updated operational data, explanation fidelity is re-evaluated to confirm prior explanations remain consistent with the updated model’s reasoning.

Absent any of these five governance layers, the AI recommendation operates as a black box in the operational workflow, regardless of how technically sophisticated its underlying model may be.

### 5.8 Additional operational use-case illustrations

Bed-management AI: A CPI-XAI implementation for inpatient bed allocation would require explanations that distinguish clinical constraint, discharge-readiness uncertainty, isolation-room availability, staffing limitations, and predicted downstream congestion. The same recommendation should be explainable differently to a bed manager, charge nurse, hospitalist, and operations executive.

Surgical scheduling AI: In perioperative operations, CPI-XAI would require explanations for why a case was prioritized, delayed, or reassigned. Explanations should expose constraints such as operating-room availability, case duration uncertainty, staffing, equipment needs, and downstream recovery-bed capacity, while preserving patient confidentiality and avoiding simplistic blame allocation.

Discharge coordination AI: A discharge-planning system should explain whether delay risk arises from clinical instability, pending laboratory results, pharmacy reconciliation, transport availability, social-care coordination, or insurance authorization. CPI-XAI would require role-aware views for case managers, physicians, nursing teams, and quality-improvement leaders.

### 5.9 Implementation constraints and change management

Implementation will face organizational, technical, cultural, and institutional constraints. Organizational barriers include unclear ownership of explanation quality, fragmented accountability across clinical operations, IT, procurement, legal, quality, and vendors, and insufficient resources for post-deployment monitoring. Technical barriers include weak interoperability, incomplete data lineage, model and explanation drift, vendor opacity, latency, dashboard overload, and difficulty connecting explanation logs to operational outcomes. Cultural barriers include automation bias, professional resistance, fear that explanations will be used punitively, inconsistent AI literacy, and disagreement over who has authority to override a recommendation.

CFIR, NASSS, and i-PARIHS suggest that implementation should be treated as facilitated organizational change rather than a one-time technical installation. A cross-disciplinary governance group should include clinical operations, nursing, quality and safety, informatics, data science, human factors, procurement, legal and compliance, workforce representatives, and patient or community voices where allocation decisions affect access or equity. Local workflow mapping, role-specific training, protected escalation routes, vendor documentation requirements, and staged pilots should precede scale-up.

The consequences of weak governance are operational as well as ethical. An opaque bed-allocation recommendation may delay transfer or disadvantage a subgroup; an unexplained staffing forecast may distribute workload inequitably or reduce trust; and a scheduling recommendation that hides capacity assumptions may worsen wait times for already underserved patients. These scenarios illustrate why explanation quality must be evaluated against workflow, fairness, safety, and accountability outcomes rather than technical plausibility alone.

Staffing-demand AI: A nurse staffing model should explain demand forecasts in terms of census volatility, acuity mix, skill mix, admission forecasts, and historical handoff patterns. A governance-ready explanation must support staffing decisions without turning uncertainty into a false appearance of precision.

The comparison in Table 1 uses five criteria: unit of analysis, implementation purpose, operational-CPI specificity, explicit explainability duties, and measurement orientation. CFIR, NASSS, i-PARIHS, and PROLIFERATE_AI primarily explain adoption, complexity, facilitation, or human-centred optimization. WHO, NIST, and FDA guidance provide broader lifecycle and normative governance expectations. CPI-XAI is more specific only in the bounded domain for which it was designed: organizational explanation governance for clinical process intelligence. It contributes five operational capabilities and a provisional scoring profile, but it has substantially less empirical validation than established implementation frameworks and authoritative guidance. CPI-XAI should therefore be used alongside, not instead of, those frameworks: implementation frameworks diagnose context and adoption; governance guidance establishes lifecycle obligations; CPI-XAI operationalizes explanation-quality questions within CPI workflows. The manuscript makes no claim of general superiority.

## 6 Proposed empirical validation roadmap

The CPI-XAI Framework is at the theoretical-constructive stage of development. It is not yet a validated measurement model. Consistent with IS framework development methodology (Gregor, 2006), we propose a three-phase empirical validation roadmap. The roadmap is intended to guide future testing and should be completed before the CPI-XAI Maturity Scale is used as a formal measurement instrument.

### 6.1 Phase 1: instrument development and content validity (6–12 months)

A candidate psychometric instrument will be developed to measure each CPI-XAI dimension as an organizational capability construct. Items should be generated deductively from the framework and reviewed by a Delphi panel of approximately 12 to 18 stakeholders, including CIOs, CMIOs, clinical operations leaders, nurse managers, quality and safety leaders, health informatics researchers, human-factors specialists, and AI governance experts. Two to three rounds should be conducted using a pre-specified consensus threshold such as at least 70% agreement and an item-level content-validity index of at least 0.78. A subsequent stakeholder workshop should test the retained items against realistic patient-flow, staffing, scheduling, and discharge scenarios, identify ambiguous language, and assess whether the required evidence is feasible to produce.

### 6.2 Phase 2: construct validity and reliability testing (12–18 months)

The instrument should be administered to approximately 200–300 respondents, or at least 10 respondents per candidate item, across academic medical centers, community hospitals, integrated delivery systems, and safety-net settings. Recruitment should be stratified across clinical operations, nursing leadership, quality and safety, health informatics, IT, AI governance, data science, and executive roles so that the scale does not reflect one professional perspective or organization type. Confirmatory factor analysis should assess CFI and TLI above 0.90, RMSEA below 0.08, and SRMR below 0.08. Reliability should be assessed using Cronbach’s alpha and McDonald’s omega, with item reduction, missing-data sensitivity analysis, and cross-role or cross-organization measurement invariance where sample size permits.

### 6.3 Phase 3: predictive validity and nomological testing (18–30 months)

Predictive validity should be tested using organizational indicators that plausibly reflect explainability governance and operational AI use. Candidate outcomes include explanation availability, audit completeness, escalation timeliness, override review quality, user trust calibration, incident-report quality, and operational performance indicators available from EHR logs, command-centre dashboards, quality and safety systems, or administrative datasets. Missing data should be examined for mechanism; low random missingness may be handled with sensitivity checks, while larger or patterned missingness should use multiple imputation or model-based approaches.

In summary, the validation roadmap treats CPI-XAI as a preliminary measurement model: Phase 1 establishes content validity, Phase 2 tests factor structure and reliability, and Phase 3 assesses whether maturity scores predict governance and operational outcomes. Until these phases are completed, the CPI-XAI Maturity Scale should be used as a conceptual and diagnostic guide, not as a validated performance metric (see Table 11).

**Table 11:** CPI-XAI empirical validation roadmap.

Phase	Activity	Method	Timeline	Key output
1	Instrument development	Delphi panel + CVI analysis	6–12 months	20-item CPI-XAI instrument
2	Construct validation	CFA + reliability analysis (*n* ≈ 200–300; ≥10 respondents per item)	12–18 months	Validated five-factor scale with reliability and fit indices
3	Predictive validity	Predictive validity testing using EHR, dashboard, quality, and administrative indicators	18–30 months	Evidence of association with governance and operational outcomes

## 7 Discussion

### 7.1 Principal findings

This structured literature review documents a substantial gap in healthcare XAI research: 175 of 187 included studies (93.6%) addressed diagnostic, disease-specific, imaging, or general clinical decision-support contexts, while operational AI governing patient flow, resource deployment, and care process management accounted for 9 studies (4.8%). The claim is descriptive and bounded by the XAI-focused search strategy: operational AI exists, but explicit explainability-governance research for operational AI remains thin.

The CPI-XAI Framework addresses the five governance gaps identified in the review (limited operational evaluation standards, role-blind design, weak causal and counterfactual support, poor workflow embedding, and absent longitudinal explanation governance) by treating XAI governance as an IS organizational capability rather than a technical property of AI models. This reframing matters because healthcare institutions govern their AI systems through policies, procurement standards, and operational protocols, not model-by-model technical audits.

### 7.2 Relationship to existing literature

The revised framework name, CPI-XAI, is intentionally used to avoid confusion with RAPIDx AI and PROLIFERATE_AI. RAPIDx AI is a specific emergency cardiac-care AI intervention evaluated across emergency departments, while PROLIFERATE_AI is a human-centred implementation and evaluation approach that assesses usability, adoption, workflow integration, barriers, optimization needs, and role-specific user experience in complex health systems. CPI-XAI differs in purpose and level of analysis: it is not an evaluation of one emergency-care tool and not a general implementation-science method. It is a governance capability framework for explainability in clinical process intelligence systems. The frameworks could be used in parallel: PROLIFERATE_AI can evaluate adoption and optimization of a health AI intervention, while CPI-XAI can specify whether explanations for operational AI are timely, role-aware, workflow-embedded, causally useful, and monitored over time (Pinero de Plaza et al., 2025; Pinero de Plaza et al., 2023).

The CPI-XAI Framework builds on but extends two bodies of work. From the XAI literature, it incorporates established explainability constructs, including fidelity, faithfulness, counterfactual availability, and local and global explanation scope, but repositions them as governance requirements rather than technical properties. From the IS literature, it applies IT Capability Theory and the DeLone–McLean model to a new domain (operational AI governance) not previously addressed by either framework’s originators.

The framework is distinct from prior XAI healthcare frameworks in one critical respect: it does not require the healthcare organization to possess AI technical expertise to apply it. The CPI-XAI dimensions and maturity levels are described in organizational capability language, specifically infrastructure, human skill, and institutional integration, that is accessible to CIOs, CMIOs, and hospital administrators responsible for AI governance decisions.

### 7.3 Implications for research

Several research directions follow. Empirical validation of the CPI-XAI instrument (detailed in Section 6) is the immediate priority. Additionally, research examining the relationship between CPI maturity and CPI-XAI maturity would establish whether organizations with more mature operational AI capability also develop stronger XAI governance. Finally, user-centered design research on role-aware explanation formats, what operational explanation granularity do different healthcare roles actually need, would address Gap 2 with primary empirical evidence.

### 7.4 Implications for practice

Economic, policy, and societal implications. CPI-XAI has implications beyond technical implementation. Economically, inadequate explanation governance can produce avoidable inefficiencies through poorly trusted operational recommendations, duplicated manual review, procurement risk, and delayed adoption of useful AI tools. Commercially, the framework can inform vendor requirements by asking suppliers to provide role-aware explanations, explanation-fidelity monitoring, audit logs, and workflow-embedded explanation access rather than generic model-transparency claims. For policymakers and accrediting organizations, CPI-XAI can be translated into accreditation questions, procurement checklists, model-governance documentation requirements, internal audit templates, and post-deployment monitoring expectations for AI used in patient flow, staffing, scheduling, and care coordination. Societally, opaque operational AI may worsen waiting times, staff workload inequity, or access disparities if bed allocation, staffing forecasts, discharge planning, or scheduling recommendations cannot be explained and challenged (Xue et al., 2023).

Practical implementation. Hospital administrators can use CPI-XAI as a preliminary readiness checklist before procurement, CIOs and CMIOs can use it to specify integration, versioning, logging, and revalidation requirements, AI governance committees can use it to evaluate whether explanation quality is monitored after deployment, and quality-improvement leaders can connect explanation failures to operational outcomes. Procurement documents and accreditation self-assessments could require role-specific explanation views, access to model and explanation change logs, documented escalation routes, subgroup testing, audit export, and evidence of explanation revalidation after updates. The framework should not be treated as a substitute for local validation, safety review, legal review, clinical governance, or formal accreditation requirements.

For hospital administrators and CIOs, CPI-XAI provides a vocabulary for XAI governance evaluation that does not require AI technical literacy. The five dimensions map onto existing IS governance and quality improvement frameworks, making them actionable in procurement decisions, vendor evaluation, and AI governance policy development.

For health IT vendors and developers, the framework specifies concrete design requirements. Operational AI systems should be assessed against CPI-XAI dimensions at procurement. Absence of role-aware explanation capability, workflow integration, or longitudinal fidelity monitoring should be treated as governance deficiencies, not minor feature gaps, and priced accordingly in procurement negotiations.

### 7.5 Limitations

Several limitations apply. First, this study is a structured literature review and framework development study, not a meta-analysis, registered scoping review, intervention-effectiveness review, risk-of-bias synthesis, or certainty-of-evidence assessment. It did not use prospective registration, formal risk-of-bias appraisal, or certainty grading. Publication bias may favor technically successful or positively evaluated XAI systems, while operational failures, abandoned implementations, and local governance practices may remain unpublished. Second, the XAI/explainability term requirement may miss relevant operational AI governance work that uses accountability, transparency, optimization, decision support, or human-factors language. The 5,913-versus-135 boundary check illustrates vocabulary sensitivity but does not quantify the number of eligible omitted studies because the broad records were not screened. Third, exclusion of non-English and grey literature may omit regional, local-language, vendor, hospital, regulator, standards, policy, procurement, and implementation materials. Fourth, the nine-study operational subset is small and heterogeneous; its coded frequencies are exploratory and cannot support strong generalization. Fifth, AI technology and governance are evolving rapidly, so the evidence map and framework may become outdated as regulation, foundation models, agentic systems, and operational deployments change; periodic search updates and versioned framework review will be necessary. Sixth, interpretive framework coding was internally reviewed but did not have a separate reliability coefficient, external Delphi panel, end-user workshop, or field pilot. Finally, the CPI-XAI Maturity Scale and XAI Operational Sovereignty concept require external content validation, psychometric testing, predictive-validity assessment, and real-world evaluation before use as validated instruments. The scoring bands, gating rules, and worked organizational assessments added in this revision are hypothetical analytical demonstrations. They are not empirical case studies, do not establish inter-rater reliability or threshold validity, and should not be used for external ranking, certification, or claims of improved clinical or operational outcomes.

## 8 Conclusion

XAI in healthcare has made genuine progress in diagnostic contexts. It has not addressed the governance needs of clinical operational AI with comparable rigor. This review documents that 9 of 187 included XAI healthcare studies (4.8%) address operational or workflow AI, while 175 studies (93.6%) address diagnostic, disease-specific, imaging, or general clinical decision-support contexts. None of the mapped operational studies proposes a CPI-specific governance framework for clinical process intelligence environments.

The CPI-XAI Governance Framework addresses this gap by mapping XAI governance requirements onto five dimensions of clinical process intelligence, grounding each dimension in established IS theory, and providing a four-level maturity model, a provisional 0–15 scoring rubric, and transparent evidence rules that healthcare organizations can pilot without requiring specialist AI expertise. The worked assessments demonstrate analytical usability, not effectiveness. The three-phase empirical validation roadmap is necessary before the framework can support benchmarking or claims about organizational performance.

The consequences of ungoverned operational AI in healthcare are real. When AI governs patient throughput, staff deployment, and care process management without traceable, role-appropriate explanations, institutions make high-stakes decisions without the governance infrastructure those decisions require. CPI-XAI is a framework designed to close that gap.

## Data Availability

The original contributions presented in the study are included in the article/Supplementary material, further inquiries can be directed to the corresponding author.
