# Supplementary material for: Beyond the black box in healthcare operations: a structured literature review and governance framework for explainable AI in clinical process intelligence systems
Source: Front Artif Intell. 2026 Oct 2;9:1864565. doi: 10.3389/frai.2026.1864565 (PMC13672608; doi:10.3389/frai.2026.1864565)
Supplement: Supplementary file 1 [file Table_1.docx]

# **Supplementary Material: Search and Screening Reporting Checklist**

*Table S1. Search and screening reporting checklist adapted from PRISMA flow-reporting items, with manuscript section cross-references.*

| **Section** | **Item #** | **Reporting item** | **Manuscript Location** |
| --- | --- | --- | --- |
| Title | 1 | Identify the report as a structured literature review and framework development study | Title page |
| Abstract | 2 | Structured summary including background, objectives, methods, results, conclusions | Abstract section |
| Introduction | 3 | Rationale for the review in context of existing knowledge | Section 1 |
| Introduction | 4 | Explicit research questions or objectives | Section 1, paragraph 5 |
| Methods – Eligibility | 5 | Inclusion and exclusion criteria and rationale | Section 3.4, Table 1 |
| Methods – Sources | 6 | Databases and other sources searched | Section 3.2 |
| Methods – Search | 7 | Full search strategy for each database | Section 3.2 |
| Methods – Selection | 8 | Study selection process, including screening stages | Section 3.3 |
| Methods – Data | 9 | Data collection process | Section 3.5 |
| Methods – Reliability | 10a | Inter-rater reliability assessment | Section 3.3 (κ = 0.84 / 0.88) |
| Methods – Statistics | 11 | Statistical methods for synthesizing results | Section 3.6 |
| Results – flow | 12 | Search and screening flow diagram | Figure 1 |
| Results – Studies | 13 | Number of studies at each selection stage with reasons | Section 4.1 and Figure 1 |
| Results – Domains | 14 | Summary statistics of study characteristics | Section 4.2, Table 2 |
| Results – Analysis | 15 | Statistical analysis results | Section 3.6; supplementary workbook |
| Discussion | 16 | Interpretation of results in context of prior evidence | Sections 7.1–7.2 |
| Discussion – Limitations | 17 | Limitations of the review | Section 7.5 |
| Discussion – Conclusions | 18 | General interpretation and implications | Section 8 |
| Registration | 24 | Protocol registration number and database | Not prospectively registered; protocol available from corresponding author |
| Competing Interests | 26 | Declaration of competing interests | Conflict of Interest section |
| Funding | 27 | Funding sources and role of funders | Conflict of Interest section |

# **Appendix A: Full Database Search Strings**

### ***PubMed / MEDLINE***

(("Explainable AI"[tiab] OR "XAI"[tiab] OR "Algorithmic Transparency"[tiab] OR "Interpretable Machine Learning"[tiab] OR "Model Explainability"[tiab]) AND ("Healthcare"[tiab] OR "Clinical"[tiab] OR "Hospital"[tiab] OR "Health System"[tiab] OR "Medical"[tiab]) AND ("Process"[tiab] OR "Operations"[tiab] OR "Workflow"[tiab] OR "Decision Support"[tiab] OR "Clinical Informatics"[tiab])) AND ("2015/01/01"[dp]:"2026/04/30"[dp])

### ***Scopus***

TITLE-ABS-KEY(("explainable AI" OR "XAI" OR "algorithmic transparency" OR "interpretable machine learning") AND ("healthcare" OR "clinical" OR "hospital") AND ("process" OR "operations" OR "workflow" OR "decision support" OR "clinical informatics")) AND PUBYEAR > 2014 AND PUBYEAR < 2027 AND DOCTYPE(ar OR re)

### ***IEEE Xplore***

("explainable AI" OR "XAI" OR "algorithmic transparency") AND ("healthcare" OR "clinical" OR "hospital") AND ("workflow" OR "clinical process" OR "decision support") Filtered: Journals only, 2015-2026

### ***Web of Science***

TS=("explainable AI" OR "XAI" OR "interpretable machine learning") AND TS=("healthcare" OR "clinical" OR "hospital") AND TS=("process" OR "workflow" OR "operations" OR "decision support") Date range: 2015-2026, Document type: Article OR Review

### ***ACM Digital Library***

[[All: "explainable ai"] OR [All: "XAI"] OR [All: "interpretable machine learning"]] AND [[All: "healthcare"] OR [All: "clinical"] OR [All: "hospital"]] Publication range: 2015-2026

### ***CINAHL***

(MH "Artificial Intelligence") AND (MH "Decision Support Systems, Clinical" OR TI "explainable AI" OR TI "XAI") AND (TI "workflow" OR TI "operations" OR TI "process improvement") Published 2015-2026

# APPENDIX B: GLOSSARY OF KEY TERMS

| **Term** | **Meaning in this manuscript** | **Why it matters** |
| --- | --- | --- |
| XAI | Explainable artificial intelligence; methods and interfaces that make AI outputs understandable to human users. | Central construct of the review and framework. |
| CPI | Clinical process intelligence; the use of data, analytics, and AI to understand and improve patient flow, resource allocation, throughput, and care coordination. | Defines the operational healthcare setting addressed by the framework. |
| CPI-XAI | The revised framework name for explainability governance in clinical process intelligence systems. | Avoids confusion with RAPIDx AI and PROLIFERATE_AI. |
| Operational AI | AI used to support workflow, staffing, scheduling, patient movement, resource deployment, or care coordination rather than diagnosis alone. | Identifies the underserved subset found in the review. |
| Role-aware explanation | An explanation tailored to a user's decision authority, workflow context, time horizon, and information needs; for example, immediate queue drivers for a charge nurse versus system-level capacity and compliance implications for an executive. | Prevents one-size-fits-all explanations and supports action without unnecessary cognitive burden. |
| Explanation fidelity | The degree to which an explanation accurately represents the model's behavior for the relevant data and remains stable or appropriately revalidated after model, data, or workflow change. | Supports version control, post-deployment monitoring, drift detection, and trustworthy revalidation. |
| IT Capability Theory | An information-systems theory linking organizational value to infrastructure, human skill, and institutional integration. | Provides one theoretical basis for treating XAI as organizational capability. |
| DeLone-McLean IS Success Model | An IS model connecting system quality, information quality, use, user satisfaction, and net benefits. | Provides evaluative constructs for CPI-XAI governance. |
